# Prolactin and growth hormone affect metaphase-II chromosomes in aging oocytes via cumulus cells using similar signaling pathways

**DOI:** 10.3389/fgene.2015.00274

**Published:** 2015-08-27

**Authors:** Irina Y. Lebedeva, Galina N. Singina, Alexander V. Lopukhov, Ekaterina N. Shedova, Natalia A. Zinovieva

**Affiliations:** Center of Animal Biotechnology and Molecular Diagnostics, L.K. Ernst Institute of Animal Husbandry, Podolsk, Moscow Region, Russia

**Keywords:** oocyte aging, chromosome modifications, cumulus cells, prolactin, growth hormone, receptors, signaling pathways

## Abstract

General senescence of the adult organism is closely connected with reproductive one. Meanwhile, the age-related reduction in the female fertility is primarily associated with a decline in the gamete quality. Molecular and cellular changes in oocytes of old mammalian females are very similar to those occurring during aging of matured ova of their young counterparts, suggesting similarities in underlying mechanisms. The aim of the present work was to study actions of two related pituitary hormones, prolactin (PRL) and growth hormone (GH), on age-associated modifications of metaphase-II (M-II) chromosomes in bovine oocytes using a model of the prolonged culture. We analyzed: (1) effects of PRL and GH on abnormal changes in the chromosome morphology in aging matured oocytes and the role of cumulus cells in these effects and (2) signaling pathways involved in the hormone actions. During the prolonged culture of oocytes, a gradual rise in the frequency of destructive modifications of M-II chromosomes was revealed. In the case of cumulus-enclosed oocytes (CEOs), PRL and GH exerted dose-dependent biphasic effects on the frequency of these modifications. Both PRL (50 ng/ml) and GH (10 ng/ml) decelerated the abnormal chromosome changes in CEOs, but did not affect the chromosome configuration in denuded oocytes. Concurrently, the presence of PRL and GH receptors in cumulus cells surrounding matured oocytes was demonstrated. Attenuating effects of both hormones on the chromosome modifications in aging CEOs were abolished by PP2 (an inhibitor of Src-family tyrosine kinases), triciribine (an inhibitor of Akt kinase), and calphostin C (a protein kinase C inhibitor). Our findings indicate that PRL and GH can exert the similar decelerating action on age-associated alterations in the M-II chromosome morphology in bovine ova, which is mediated by cumulus cells and may be related to activation of Src-family tyrosine kinases as well as Akt- and protein kinase C-dependent signal pathways.

## Introduction

General senescence of the adult organism is closely connected with reproductive one. It is well established that the female reproductive performance, primarily the ovarian function, deteriorates with age in many vertebrates including humans, with underlying fundamental mechanisms being conserved among species (Finch and Holmes, 2010; Ottinger, 2010). In mammals, an initial reduction in the female fertility is primarily associated with a decline in the gamete fitness (Tatone et al., 2008). The impairment of the oocyte quality is usually thought to be caused by age-dependent changes in general and specific functions of both oocytes and their nursing somatic follicular cells (Broekmans et al., 2007; Tatone et al., 2008; Tatone and Amicarelli, 2013). Reproductive (or maternal) aging proceeds slowly and involves mainly immature oocytes arrested at the diplotene stage.

Postovulatory aging of mature mammalian oocytes is another biological phenomenon leading to an age-related decline in the quality of female gametes. This type of senescence occurs quickly (within 1–2 days) in ova arrested at the metaphase-II (M-II) stage in the absence of activation stimuli (Miao et al., 2009). Reproductive aging and postovulatory aging are characterized by similar functional changes of oocytes including chromosome abnormalities, spindle defects, disturbances in segregation of homologous chromosomes or sister chromatids, an increased predisposition to apoptosis and parthenogenesis, a decreased fertilizability and developmental capacity (Battaglia et al., 1996; Wu et al., 2000; te Velde and Pearson, 2002; Ma et al., 2005; Tatone et al., 2006; Miao et al., 2009). According to the current state of knowledge, molecular transformations occurring in the oocyte cytoplasm underlie the above listed negative functional changes. These transformations are primarily related to alterations in the pattern of synthesis and post-translational modifications of effector proteins and to disturbances of the mitochondrial function and calcium homeostasis (Eichenlaub-Ritter et al., 2004; Tatone et al., 2006, 2008; Miao et al., 2009; McReynolds et al., 2012). In particular, some functional changes associated with oocyte aging have been shown to be related to the compromised function of intra-oocyte signaling pathways dependent on different protein kinases including MAP kinase, protein kinase C, and Akt (Tatone et al., 2006; Petr et al., 2011; Cecconi et al., 2014; McGinnis et al., 2014). Meanwhile, cellular and molecular changes in postovulatory ova aging *in vivo* are very similar to those occurring during *in vitro* aging of *in vivo* and *in vitro* matured oocytes, suggesting similarities in the underlying mechanisms (Miao et al., 2005, 2009). Thus the prolonged culture of mature mammalian oocytes is a convenient model for the comprehensive study of physiological factors and signal systems involved in regulation of the oocyte senescence.

The available evidence points to participation of two closely-related hormones, prolactin (PRL) and growth hormone (GH), in modulation of the mammalian oocyte maturation and developmental competence (Izadyar et al., 1996; Bole-Feysot et al., 1998; Hull and Harvey, 2002; Lebedeva et al., 2014b). Receptors of PRL and GH or their mRNA have been detected in oocytes and surrounding cumulus cells of different species including cows (Bevers and Izadyar, 2002; Marchal et al., 2003; Picazo et al., 2004; Lebedeva et al., 2014b). Both hormones can modulate the mitochondrial activity and/or calcium homeostasis in bovine oocytes maturing *in vitro* (Kuzmina et al., 1999, 2007), indicating the hormonal implication in processes modified by aging. Furthermore, in various types of mammalian cells, PRL and GH are able to activate signal cascades dependent on MAP kinase, protein kinase C, and Akt (Postel-Vinay and Finidori, 1995; Bole-Feysot et al., 1998; Di Rosa et al., 2009; Devesa et al., 2014), which in turn are involved in regulation of some functional changes in aging oocytes. Mammalian follicular fluid is known to contain both hormones derived from the circulation as well as produced locally by ovarian cells (Borromeo et al., 1998; Mendoza et al., 2002; Modina et al., 2007; Marano and Ben-Jonathan, 2014). Immediately after ovulation, follicular fluid carrying the ovum becomes the major component of tubal fluid (Lyons et al., 2006). Thus, there are reasons to assume that PRL and GH may act at least temporarily within the oviduct and affect aging processes in mature oocytes.

To date, little is known about physiological factors regulating the speed of oocyte senescence. Using the nematode *Caenorhabditis elegans* as the justified model of female reproductive aging, it has been recently demonstrated that two conserved endocrine/growth factor pathways, the insulin/insulin-like growth factor-1 (IGF-1) and transforming growth factor-β (TGF-β) pathways, act in various somatic tissues to control oocyte aging (Luo et al., 2010). According to the current concept, similar somatic signals might also regulate the oocyte quality in older women (Ellis and Wei, 2010). This concept is supported by data for age-related changes in the expression of some genes associated with the insulin/IGF-1 and TGF-β pathways in human cumulus cells (Al-Edani et al., 2014). Interactions between gametes and somatic cells are of considerable importance in the case of postovulatory aging as well (Miao et al., 2009). During *in vitro* aging of mammalian oocytes, both accelerating and decelerating effects of cumulus cells on different negative functional changes in mature ova have been found (Miao et al., 2005; Takahashi et al., 2009; Wu et al., 2011). Furthermore, the impaired expression of several genes related to the mitochondrial function, metabolism, apoptosis, and the antioxidant defense in cumulus cells surrounding *in vitro* aging goat oocytes has been revealed (Zhang et al., 2013). However, physiological regulators and signal systems involved in cumulus-oocyte interactions determining the matured ovum senescence are still not clearly understood.

We have previously found abnormal modifications of the chromosome morphology in bovine M-II oocytes aging *in vitro*, with the surrounding cumulus investment promoting these negative processes (Lebedeva et al., 2014a). Since PRL and GH at certain concentrations could exert inhibitory effects mediated by cumulus/granulosa cells on destructive changes of chromosomes in bovine oocytes maturing *in vitro* (Kuzmina et al., 1999; Lebedeva et al., 2005), one would expect similar hormonal effects on the senescent oocytes. Therefore, the present study was conducted to test a hypothesis that PRL and GH are able to suppress the M-II chromosome aberrations in aging bovine oocytes by transmitting signals through somatic cumulus cells. To attain this aim we analyzed: (1) effects of PRL and GH on abnormal changes in the chromosome morphology in aging mature oocytes and the role of cumulus cells in these effects and (2) signaling pathways involved in the hormonal effects. In addition, the presence of PRL and GH receptors in cumulus cells surrounding matured oocytes has been verified, because cumulus expression of different proteins including hormonal receptors is dramatically reduced in the course of oocyte maturation (Devjak et al., 2012). The choice of tested signaling pathways dependent on Akt, protein kinase C, and MAP kinase was due to their implication in both oocyte and somatic aging (Bitar, 2003; Tatone et al., 2006; Salminen and Kaarniranta, 2010; Petr et al., 2011; Cecconi et al., 2014). To assess the participation of the previously mentioned protein kinases in actions of PRL and GH on destructive changes of M-II chromosomes, the influence of triciribine (an inhibitor of Akt kinase), calphostin C (a protein kinase C inhibitor), and U0126 (a MEK 1/2 inhibitor) on these actions was examined. Furthermore, the respective involvement of Src-family tyrosine kinases associated constitutively with PRL and GH receptors (Waters and Brooks, 2011; Martín-Pérez et al., 2015) was tested using genistein (a non-selective inhibitor of tyrosine kinases) and PP2 (an inhibitor of Src-family tyrosine kinases).

## Materials and Methods

Unless otherwise stated, all media and chemicals were purchased from Sigma-Aldrich Chemical Corporation (St. Louis, MO, USA).

### Oocyte Collection, Handling, and *in Vitro* Maturation

Slaughterhouse-derived bovine ovaries were transported to the laboratory in a thermo box with sterile saline at 30–35°C, and cumulus-enclosed oocytes (CEOs) were obtained by wall dissection of 2–8 mm antral follicles. The oocyte retrieval and handling were performed in a wash medium consisting of HEPES-buffered TCM-199 containing 5% (v/v) fetal calf serum (FCS; Hyclone Laboratories, Logan, UT, USA) and 50 μg/mL of gentamicin sulfate. The CEOs were washed twice in the wash medium and selected under a stereomicroscope. Only oocytes with a complete, compact, multilayer cumulus and finely granulated homogenous ooplasm were used for the study. Groups of CEOs were matured for 20 h in 500 μL of a maturation medium at 38.5°C under 5% CO_2_ in humidified air. The following maturation medium was used: HEPES-buffered TCM-199 (with Earle’s salts and L-glutamine), containing 0.2 mM sodium pyruvate, 50 μg/mL of gentamicin, and 10% (v/v) FCS, supplemented with 10 μg/mL of porcine follicle-stimulating hormone and 10 μg/mL of ovine luteinizing hormone.

### Design of Oocyte Prolonged Culture Experiments

After 20 h of maturation, most of CEOs were immediately used for the prolonged culture. Another portion of the oocytes was denuded of their cumulus cells by incubating the CEOs in the above mentioned wash media containing 0.1% (v/v) hyaluronidase for 1 min at 37°C and subsequent gentle pipetting through a fine needle pipette (with the hole diameter of 130 μm). The denuded oocytes (DOs) were washed twice from hyaluronidase and examined under an inverted light microscope (at magnification × 200) to ensure the complete removal of cumulus cells. Thereafter, CEOs or DOs were transferred to an aging medium consisting of HEPES-buffered TCM-199 supplemented with 0.2 mM sodium pyruvate, 50 μg/mL of gentamicin, and 10% (v/v) FCS (Control) and cultured at 38.5°C under 5% CO_2_ in humidified air. In experimental groups, either pituitary bovine PRL (20 IU/mg; Research Center for Endocrinology, Moscow, Russia) or recombinant bovine GH (Monsanto, St. Louis, MO, USA) was added to the aging medium. In dose-dependent experiments, two different preparations of bovine PRL (Research Center for Endocrinology, Moscow, Russia and USDA bPRL B-1, Beltsville, MD, USA) were used for comparison. Frozen aliquots of a stock solution (50 μg/mL of PRL or 10 μg/mL of GH in saline) were diluted by the aging medium immediately prior to culture.

In the first experiment, CEOs were cultured for 24 h in the aging medium containing different concentrations of either PRL (0, 20, 50, 150, and 500 ng/mL), or GH (0, 2.5, 5, 10, 20, and 50 ng/mL), or recombinant human epidermal growth factor (EGF; 0, 0.2, 1, 10, 50, and 500 ng/mL; Thermo Fisher Scientific, Waltham, MA, USA). In the second experiment, CEOs and DOs were incubated for 12, 24, 36, or 48 h with and without 50 ng/mL of PRL or 10 ng/mL of GH. In the third experiment, CEOs were cultured for 24 h in the absence and in the presence of either PRL (50 ng/mL) or GH (10 ng/mL) and/or protein kinase inhibitors. The following inhibitors were applied: (1) genistein, the non-selective inhibitor of tyrosine kinases (40 μM; ICN Biomedicals, Aurora, OH, USA), (2) PP2, the inhibitor of Src-family tyrosine kinases (20 μM), (3) triciribine, the inhibitor of Akt kinase (50 μM), (4) calphostin C, the protein kinase C inhibitor (1 μM; Calbiochem, Darmstadt, Germany), and (5) U0126, the MEK 1/2 inhibitor (20 μM; Promega, Madison, WI, USA). At the end of culture, all oocytes were fixed to determine their nuclear status. In all the experiments, the proportion of oocytes at the M-II stage was no less than 72%.

### Assessment of Oocyte Nuclear Material

To evaluate nuclear maturation and the M-II chromosome morphology in oocytes, cytogenetic preparations were performed by the method of Tarkowski (1966) with some modifications (Kuzmina et al., 1999). The state of the nuclear material was examined under a light microscope (Opton, Germany) at magnification × 1000 using criteria described earlier (Ernst et al., 1980; Homa, 1988). The following morphological abnormalities were ascribed to destructive changes of M-II chromosomes: (1) decondensation (a loss of clear morphological contours, an increase in the chromosome volume and/or uneven morphological contours), (2) chromosome decondensation and/or partial adherence, (3) chromosome clumping into a single mass, and (4) fragmentation.

### Immunocytochemical Analysis

The protein expression of PRL and GH receptors in cumulus cells surrounding *in vitro* matured oocytes was detected by immunocytochemistry as described previously for freshly isolated bovine CEOs (Lebedeva et al., 2014b). Briefly, following 20 h of maturation, CEOs were washed twice in PBS containing 0.2% (w/v) bovine serum albumin (PBS-BSA) and fixed with 4% (w/v) paraformaldehyde in PBS for 15 min. After washing, the specimens were permeabilized for 30 min with 0.1% (v/v) Triton X-100 in PBS-BSA (in the case of the PRL receptor) or with 0.5% (v/v) Triton X-100 in PBS-BSA (in the case of the GH receptor). Nonspecific binding was blocked by incubating the CEOs with 10% (v/v) goat serum (Vector Laboratories, Burlingame, CA, USA) in PBS containing 1% (w/v) BSA for 1 h at room temperature. For detection of PRL and GH receptors, the specimens were incubated respectively with mouse anti-PRL receptor monoclonal antibody MA1-610 (Thermo Scientific, Rockford, IL, USA; 1:50 dilution) or mouse anti-GH receptor monoclonal antibody MAB 263 (Abcam, Cambridge, MA, USA; 1:50 dilution) overnight at 4°C. The primary antibody MA1-610 reacts both with long and short PRL receptor isoforms and detects the receptor in tissues of different mammalian species including bovine cumulus cells (Lebedeva et al., 2014b). The antibody MAB 263 has been extensively validated for immunohistochemical studies in various types of tissues including bovine ovary (Kölle et al., 1998). Thereafter, the CEOs were incubated with biotinylated goat anti-mouse antibody (Vector Laboratories, Burlingame, CA, USA; 1:500 dilution) for 30 min at room temperature. All antibodies were diluted in PBS containing 1% (w/v) BSA and 3% (v/v) goat serum. For visualization of the specific staining, Vectastain ABC reagent and 3-amino-9-ethylcarbazole (AEC) substrate (all purchased from Vector Laboratories, Burlingame, CA, USA) were applied. The CEOs were counterstained by hematoxylin and mounted in a droplet of the glycerol-PBS mixture (1:3, v/v). All specimens were evaluated for the presence of PRL and GH receptors using the light microscope at magnification × 400.

Specificity of the immunodetection was proved by several negative controls: (1) omission of the first antibody, (2) omission of the secondary antibody, and (3) incubation with AEC alone to show the absence of endogenous peroxidases. Layers of freshly isolated bovine membrana granulosa, expressing PRL and GH receptors (Kölle et al., 1998; Lebedeva et al., 2001), were employed as a positive control. A total of 24 and 41 CEOs were used for immunocytochemical detection of PRL and GH receptors, respectively.

### Statistical Analysis

All treatments in culture experiments were repeated 3–6 times. The numbers of oocytes used per each treatment are indicated in figure legends. Results were expressed as means ± SEM. Data were analyzed by one-way or two-way ANOVA followed by the Tukey’s HSD test using SigmaStat software package. If the data expressed as percentages did not meet the assumption of normal distribution or homogeneity of variance, they were arcsine transformed before analysis. In the case of two-way ANOVA, the statistical model included the main effects and all interactions. Independent variables were PRL or GH treatments and the aging duration or the inhibitor treatment. A probability of *p* < 0.05 was considered to be statistically significant.

## Results

### Effects of PRL and GH on M-II Chromosomes During *in Vitro* Aging of Bovine Cumulus-Enclosed and Denuded Oocytes

**FIGURE 1:**
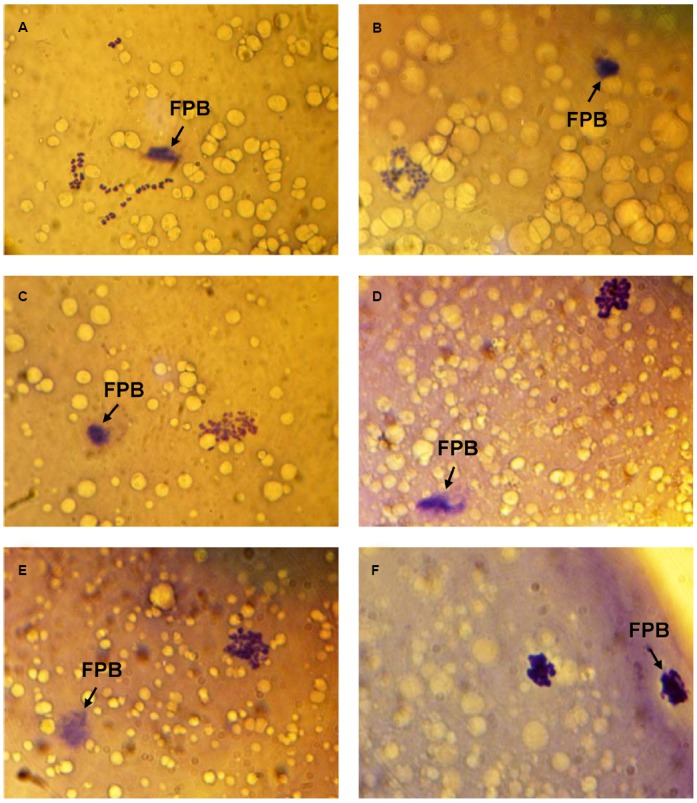
**Morphology of M-II chromosomes in aging bovine oocytes. (A)** Chromosomes without signs of abnormal changes, **(B)** Chromosome decondensation (a loss of clear morphological contours), **(C)** Chromosome decondensation (unclear and uneven morphological contours), **(D)** Chromosome decondensation (uneven morphological contours and an increase in the chromosome volume), **(E)** Chromosome decondensation (unclear and uneven morphological contours) and partial adherence, **(F)** Chromosome clumping into a single mass. Black arrow indicates the first polar body (FPB). Original magnification: × 1000.

Immediately after *in vitro* maturation, the proportion of M-II oocytes with signs of chromosome abnormalities did not exceed 20% in all experiments. Following 24 h of the prolonged culture in the control aging medium, the rate of CEOs with destructive modifications of M-II chromosomes increased up to 50–64%. Meanwhile, the chromosome decondensation was the most common abnormal change that was manifested by various morphological signs (Figure 1). At the same time the chromosome fragmentation was observed very seldom.

**FIGURE 2:**
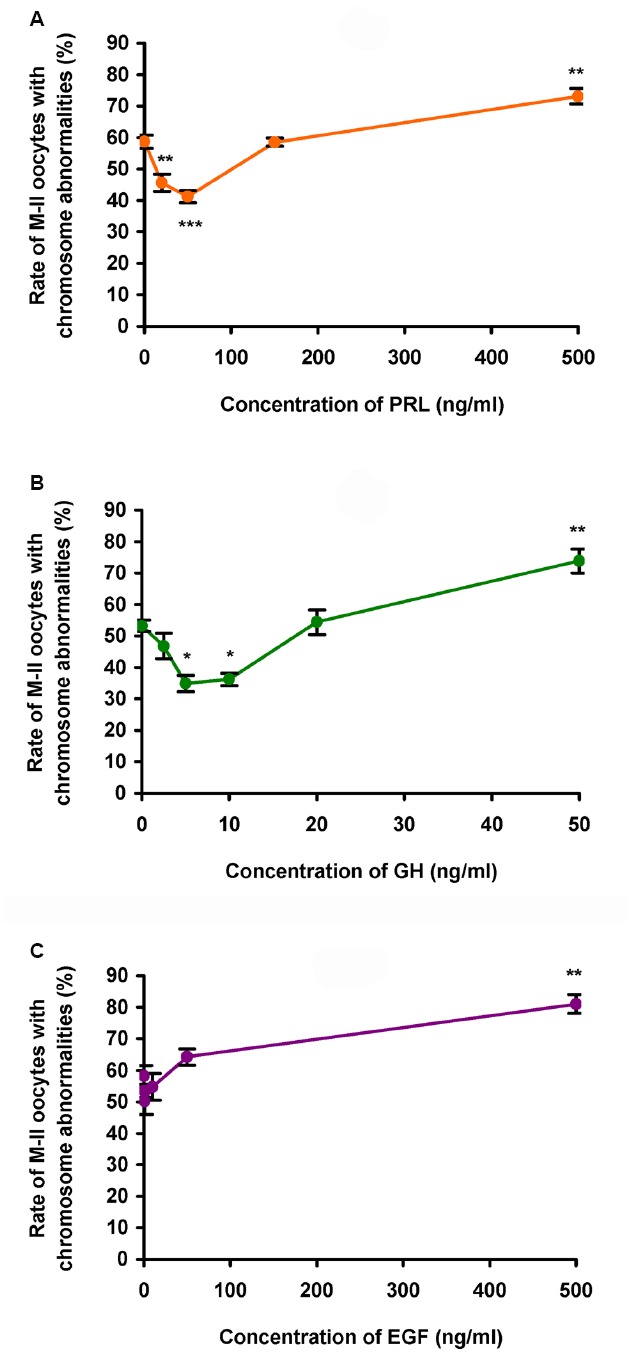
**Effects of different concentrations of PRL (A), GH (B), and EGF (C) on destructive changes in the M-II chromosome morphology during 24 h aging of bovine cumulus-enclosed oocytes.** Data represent means ± SEM of 3–6 replicates using 51–91 oocytes per treatment. *p < 0.05, **p < 0.01, ***p < 0.001 compared with the respective groups without PRL, GH, or EGF.

During 24 h aging of CEOs, PRL and GH exerted dose-dependent biphasic effects on the frequency of abnormal chromosome modifications. Since the data obtained for both PRL preparations were identical, they were combined to produce a single dose-dependent curve. The application of PRL at concentrations of 20–50 ng/mL caused a decline in the frequency of M-II chromosome modifications (at least *p* < 0.01), with the maximum reducing effect being observed at a concentration of 50 ng/mL (Figure 2A). By contrast, at a concentration of 500 ng/mL, PRL enhanced destructive changes in the M-II chromosome morphology (*p* < 0.01). The pattern of the dose-dependent curve for GH was very similar to that for PRL (Figure 2B). As compared to the control medium, the rate of oocytes with abnormal chromosome changes was reduced in the presence of 5–10 ng/mL of GH (*p* < 0.05) and increased in the presence of 50 ng/mL of GH (*p* < 0.01). At the same time EGF, another well-known modulator of oocyte maturation (Conti et al., 2006), did not inhibit destructive modifications of M-II chromosomes at all concentrations tested (Figure 2C). So, in further experiments, 50 ng/mL of PRL and 10 ng/mL of GH were used for characterization of the suppressive influence of the hormones on age-associated chromosome changes in bovine oocytes.

**FIGURE 3:**
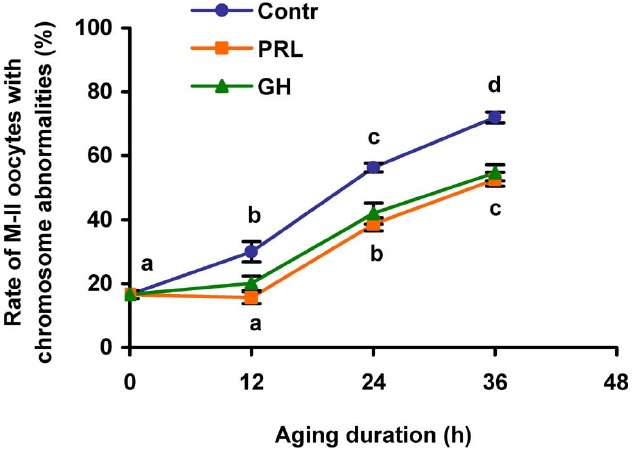
**Effects of PRL (50 ng/mL) and GH (10 ng/ml) on destructive changes in the M-II chromosome morphology during aging of bovine cumulus-enclosed oocytes.** Data represent means ± SEM of 4–5 replicates using 77–101 oocytes per treatment. Means with different letters differ significantly (at least *p* < 0.05).

In the course of the prolonged culture of CEOs in the control medium, a rise in the rate of the oocytes with destructive changes of the chromosome configuration occurred by 12 h of aging (*p* < 0.05) and persisted up to 36 h (*p* < 0.001; Figure 3). The addition of PRL (50 ng/mL) or GH (10 ng/mL) to the aging medium resulted in a decrease of this rate throughout the culture period (at least *p* < 0.05). Thus, the frequency of abnormal modifications of M-II chromosomes in aging oocytes increased more slowly in the hormone-treated groups than in the control group.

**FIGURE 4:**
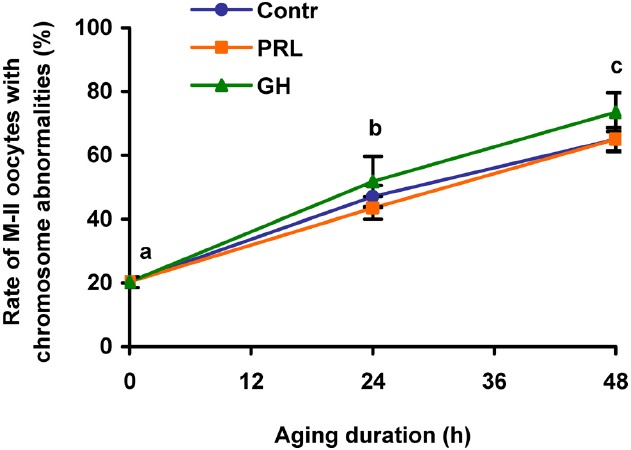
**Destructive changes in the M-II chromosome morphology during aging of bovine cumulus-free oocytes in the presence and in the absence of PRL (50 ng/ml) or GH (10 ng/ml).** Data represent means ± SEM of 4–5 replicates using 83–104 oocytes per treatment. Means with different letters differ significantly (at least *p* < 0.05).

Effects of PRL and GH on destructive changes of M-II chromosomes in DOs were explored after 24 and 48 h of aging, since the removal of cumulus cells had been shown to decelerate these changes in bovine oocytes (Lebedeva et al., 2014a). It was found that the gradual rise in the rate of DOs with chromosome abnormalities during the prolonged culture was unaffected by both PRL and GH (Figure 4). Therefore, only CEOs were used in the subsequent culture experiments to study signaling pathways involved in the hormone actions.

### Immunocytochemical Localization of PRL and GH Receptors in Bovine Cumulus Cells Surrounding Matured Oocytes

**FIGURE 5:**
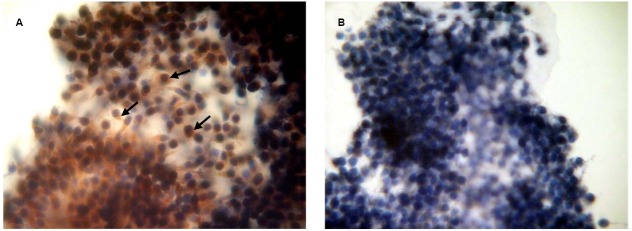
**Immunocytochemical detection of PRL receptors in cumulus cells after 20 h maturation of bovine cumulus-enclosed oocytes.** Specific localizations were detected by MA1-610 antibody and the red 3-amino-9-ethylcarbazole (AEC) chromophore. Nuclei were counterstained with hematoxylin. **(A)** Positive staining. Black arrows indicate PRL receptor-specific immunoreaction. **(B)** Negative control performed by omitting the primary antibody. Original magnification: × 400.

**FIGURE 6:**
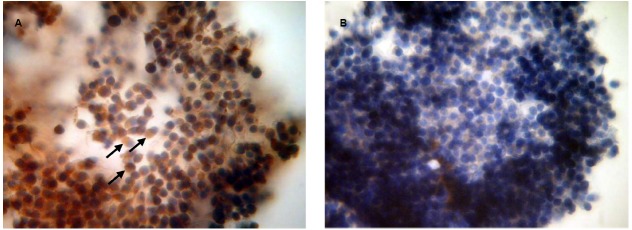
**Immunocytochemical detection of GH receptors in cumulus cells after 20 h maturation of bovine cumulus-enclosed oocytes.** Specific localizations were detected by MAB 263 antibody and the red 3-amino-9-ethylcarbazole (AEC) chromophore. Nuclei were counterstained with hematoxylin. **(A)** Positive staining. Black arrows indicate GH receptor-specific immunoreaction. **(B)** Negative control performed by omitting the primary antibody. Original magnification: × 400.

To confirm the availability of cumulus-mediated pathways of PRL and GH signaling into M-II oocytes, the presence of the respective receptors in cumulus cells following 20 h maturation of CEOs was examined. Receptors of PRL were detected in the cells of all bovine cumulus-oocyte complexes tested using MA1-610 antibody and the red AEC-chromophore (Figure 5A). No specific immunoreactivity was found in negative controls performed by omitting the primary antibody (Figure 5B). Furthermore, most of cumulus cells surrounding *in vitro* matured oocytes showed the intensive red staining for anti-GH receptor antibody MAB 263 (Figure 6A), whereas the specific immunoreactivity was not present in the negative controls (Figure 6B). Layers of freshly isolated membrana granulosa (positive control) containing PRL and GH receptors (Kölle et al., 1998; Lebedeva et al., 2001) also demonstrated the red staining (data not shown). Concurrently, CEOs incubated without the secondary antibody or with AEC alone did not show the specific staining.

### Signaling Pathways Involved in PRL and GH Modulation Of M-II Chromosome Abnormalities in Aging Bovine Cumulus-Enclosed Oocytes

An involvement of different protein kinases in PRL and GH signaling into aging CEOs was studied by testing effects of inhibitors of tyrosine kinases, Akt kinase, protein kinase C, and MEK 1/2 on the decelerating hormonal influence on the chromosome modifications. The used concentrations of genistein (40 μM), PP2 (20 μM), and U0126 (20 μM) were very close to the respective concentrations, which were effective in suppressing PRL and GH actions on different mammalian cells (Fresno Vara et al., 2001; Huang et al., 2003; Gutzman et al., 2004; Zhang et al., 2004, 2006; Lebedeva et al., 2011). Concentrations of triciribine (50 μM) and calphostin C (1 μM) were chosen on the basis of the published values for the respective IC50 (Tamaoki and Nakano, 1990; Gürsel et al., 2011).

**FIGURE 7:**
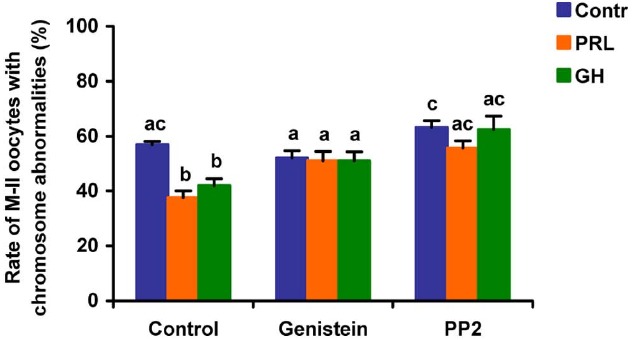
**Effects of genistein (40 μM), the non-selective inhibitor of tyrosine kinases, and PP2 (20 μM), the inhibitor of Src-family tyrosine kinases, on destructive changes in the M-II chromosome morphology during 24 h aging of bovine cumulus-enclosed oocytes in the presence and in the absence of PRL (50 ng/ml) or GH (10 ng/ml).** Data represent means ± SEM of 4 replicates using 82–96 oocytes per treatment. Means with different letters differ significantly (at least *p* < 0.05).

**FIGURE 8:**
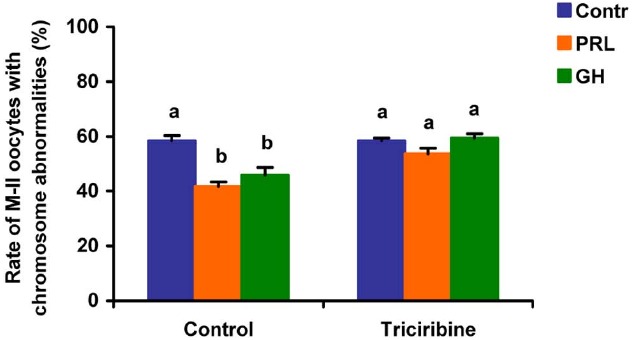
**Effects of triciribine (50 μM), the inhibitor of Akt kinase, on destructive changes in the M-II chromosome morphology during 24 h aging of bovine cumulus-enclosed oocytes in the presence and in the absence of PRL (50 ng/ml) or GH (10 ng/ml).** Data represent means ± SEM of 4 replicates using 74–92 oocytes per treatment. Means with different letters differ significantly (at least *p* < 0.05).

**FIGURE 9:**
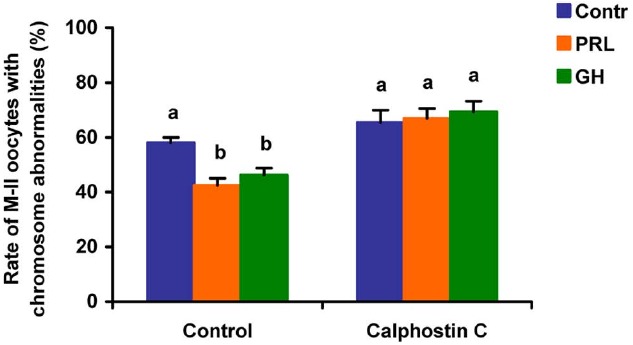
**Effects of calphostin C (1 μM), the inhibitor of protein kinase C, on destructive changes in the M-II chromosome morphology during 24 h aging of bovine cumulus-enclosed oocytes in the presence and in the absence of PRL (50 ng/ml) or GH (10 ng/ml).** Data represent means ± SEM of 4 replicates using 73–81 oocytes per treatment. Means with different letters differ significantly (at least *p* < 0.05).

**FIGURE 10:**
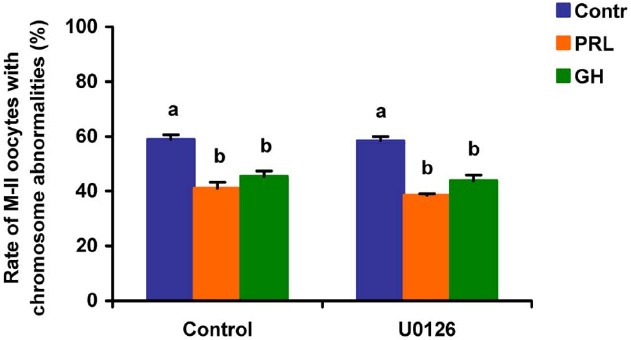
**Effects of U0126 (20 μM), the MEK 1/2 inhibitor, on destructive changes in the M-II chromosome morphology during 24 h aging of bovine cumulus-enclosed oocytes in the presence and in the absence of PRL (50 ng/ml) or GH (10 ng/ml).** Data represent means ± SEM of 4 replicates using 73–86 oocytes per treatment. Means with different letters differ significantly (at least *p* < 0.05).

When added to the aging medium, genistein, the non-selective inhibitor of tyrosine kinases, and PP2, the inhibitor of Src-family tyrosine kinases, eliminated the revealed effects of PRL and GH on the chromosome destruction (Figure 7). Meanwhile, a rise in the rate of CEOs with abnormal modifications of M-II chromosomes was more pronounced in the presence of PP2 (*p* < 0.001) than genistein (*p* < 0.05). Triciribine, the inhibitor of Akt kinase, and calphostin C, the protein kinase C inhibitor, also increased the frequency of chromosome modifications (*p* < 0.001) during the prolonged culture of CEOs in the medium containing PRL or GH (Figures 8 and 9). At the same time the hormonal action on the chromosome destruction in the aging oocytes was unchanged in the presence of U0126, the MEK 1/2 inhibitor (Figure 10). Concurrently, at concentrations used, all the inhibitors did not affect the frequency of chromosome abnormalities in the respective control groups.

## Discussion

The present research has been directed toward elucidating the pattern and mechanisms of action of two closely-related pituitary hormones, PRL and GH, on abnormal modifications of M-II chromosomes in matured bovine oocytes aging *in vitro*. The data obtained indicate for the first time that both hormones are able to eliminate the accelerating effect of surrounding cumulus cells on aging processes associated with chromosome changes in oocytes. In this case PRL and GH can use similar pathways, acting through cumulus cells expressing the respective receptors and activating signaling cascades dependent on Src-family tyrosine kinases, Akt kinase, and protein kinase C.

In line with our earlier findings (Lebedeva et al., 2014a), in the current study, the prolonged culture of matured bovine oocytes was accompanied by different modifications in the morphology of M-II chromosomes, with the most common abnormal change being their decondensation or decondensation coupled with adherence. Similar chromosome aberrations have been found in human oocytes aged *in vitro* for 3–4 days (Eichenlaub-Ritter et al., 1988). Furthermore, using quantitative 3D analysis of nuclear material in oocytes of aging mice, Tian et al. (2013) have recently demonstrated an enhancement of adhesion between chromosomes in metaphase-I that may be a consequence of their decondensation and the decrease of interchromosomal distance. Thus, a decline in the degree of metaphase chromosome condensation is likely to be one of the functional nuclear alterations associated with M-II oocyte senescence. It might result in the incomplete silencing of gene transcription and subsequent abnormalities in the embryo/fetus development, which are observed following fertilization of aged oocytes (Miao et al., 2009). One can also speculate that destructive changes in the M-II chromosomal morphology are induced by epigenetic modifications driving chromosome transformations during meiosis (Endo et al., 2005; Ivanovska and Orr-Weaver, 2006). To date, various epigenetic changes, mainly a decline in the methylation level of DNA and histones and a rise in the level of histone acetylation, have been revealed in the course of mammalian oocyte aging (Huang et al., 2007; Liang et al., 2008; Manosalva and González, 2010). Furthermore, a decrease in the histone methylation level in oocytes of old female mice has been shown to be associated with different structural chromosomal abnormalities including decondensation (Manosalva and González, 2010).

Our data have demonstrated that both PRL and GH affect the morphology of M-II chromosomes in CEOs in a biphasic dose-dependent manner, which is typical for actions of these hormones in different cell systems (Ilondo et al., 1994; Hodson et al., 2010; Lebedeva et al., 2014b). A bell-shaped pattern of dose-response curves for PRL and GH is usually attributed to the two-site mechanism of the hormone binding to their receptors involving dimerization of these latter and self-antagonism at high concentrations when monomeric hormone-receptor complexes become predominant (Kelly et al., 1994). However, this theoretical model cannot explain the opposite pattern of effects of low and high hormonal concentrations found in the present study, suggesting an implication of additional factors. Dose-response experiments have revealed inhibitory effects of low concentrations of PRL (20–50 ng/ml) and GH (5–10 ng/ml) but not EGF on abnormal modifications of M-II chromosomes in aging bovine oocytes. These concentrations are the same as concentrations, which have been effective at stimulating *in vitro* the nuclear maturation or developmental capacity of bovine CEOs (Kuzmina et al., 2001, 2007). Moreover, they are very close to the respective hormonal concentrations found in the bovine plasma and follicular fluid (Wise and Maurer, 1994; Borromeo et al., 1998; Modina et al., 2007). Physiological levels of intratubular PRL and GH possibly derived from the circulation are presently unknown; however, follicular fluid containing both hormones flows into the oviduct following ovulation (Lyons et al., 2006), permitting their local effects. The possibility for PRL and GH actions within the oviduct is also supported by evidence for the expression of the respective receptors in mammalian oviductal cells (Shao et al., 2008; Steffl et al., 2009). It should be emphasized that concentrations of PRL and GH enhancing destructive changes of M-II chromosomes are 10 to 20 times higher than physiological ones. This fact gives grounds to expect an inhibitory influence of PRL and GH on the metaphase chromosome modifications during postovulatory aging of matured bovine oocytes.

In mammals, cumulus cells are vitally important for regulating processes of oocyte maturation, ovulation, and fertilization (Tanghe et al., 2002). However, their function in oocyte aging is not evident, since they can play both positive and negative roles in maintaining the quality of the senescent ovum (Miao et al., 2005; Takahashi et al., 2009; Wu et al., 2011). Furthermore, it has been demonstrated that cumulus cells surrounding aging mouse and porcine oocytes are exposed to apoptosis and, in turn, begin to produce soluble substances, which accelerate oocyte aging in a paracrine manner (Wu et al., 2011; Zhu et al., 2015). We have previously shown that bovine cumulus cells surrounding aging oocytes are undergone apoptotic degeneration and their removal lead to delaying the abnormal changes of M-II chromosomes, suggesting an accelerating effect of the somatic investment on these changes (Lebedeva et al., 2014a). In the present work, both PRL and GH at low concentrations decelerated aging processes associated with chromosome modifications in CEOs, exerting an effect much like that of the cumulus removal. Meanwhile, PRL and GH did not affect the chromosomal aberrations in cumulus-free oocytes, implying that the hormonal impacts were achieved through cumulus cells. The availability of cumulus-mediated pathways of the hormone actions was further confirmed by the immunocytochemical localization of PRL and GH receptors in cumulus cells surrounding *in vitro* matured bovine oocytes. Taking into account all these data, one can assume that bovine cumulus cells may produce aging-promoting factor/factors (APF) contributing to abnormal modifications of oocyte chromosomes, while PRL and GH are able to suppress the production or action of APF.

Finally, the implication of the relevant protein kinases into the actions of PRL and GH on aging bovine CEOs was examined using inhibitors of the respective signaling pathways. It has been found that inactivation of Src-family tyrosine kinases, Akt kinase, and protein kinase C results in blocking of the decelerating action of the studied hormones on abnormal modifications of M-II chromosomes. By contrast, the inhibition of the MEK1/2 activity did not abolish the hormonal effects on the chromosome aberrations. Thus, the effects of PRL and GH were attained by activating the similar signaling cascades dependent on Src-family tyrosine kinases, Akt kinase, and protein kinase C. It should be noted that, in the absence of the hormones, inhibitors of these intracellular pathways did not affect the chromosome destruction, suggesting a low basal activity of the respective protein kinases in aging cumulus-oocyte complexes. The reduced activity of the signaling pathways might be a consequence of the impaired functional status of cumulus cells surrounding aging oocytes that had been previously demonstrated (Zhang et al., 2013; Zhu et al., 2015).

**FIGURE 11:**
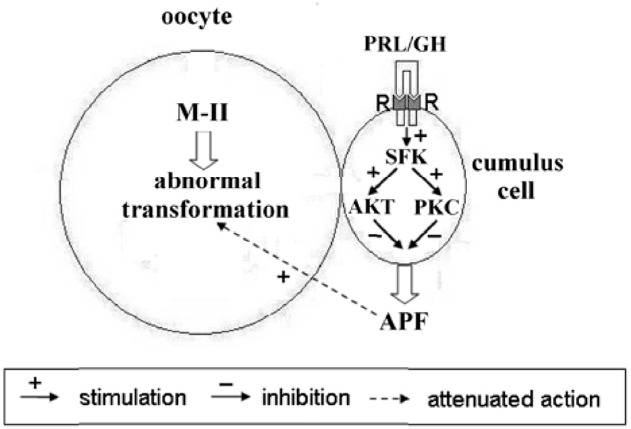
**Schematic representation of possible mechanisms of PRL and GH actions on abnormal modifications of M-II chromosomes in aging bovine oocytes.** The hormone binding to the respective receptors (R) on cumulus cells causes an activation of receptor-associated Src-family kinases (SFK), which control signaling pathways dependent on Akt and protein kinase C (PKC). These events result in inhibiting the production of the cumulus-derived aging-promoting factor (APF) that accelerates abnormal chromosome modifications.

The revealed similarity in mechanisms of PRL and GH actions on senescent oocytes is obviously due to the close relationship both between the hormones and between their receptors (Kelly et al., 1994). Although JAK2/STAT (Janus kinase 2/Signal transducers and activators of transcription) is the main signal pathway activated in response to PRL and GH, other pathways are also involved in their effects. To date, it has been well established that Src-family tyrosine kinases are associated constitutively with PRL and GH receptors and can control activation of different signaling cascades including PI3K/Akt and MEK/ERK (Barclay et al., 2010; Waters and Brooks, 2011; Martín-Pérez et al., 2015). In its turn, the PI3K/Akt pathway may be connected with protein kinase C dependent pathway through activation of phospholipase C and generation of diacylglycerol (Divecha and Irvine, 1995). Meanwhile, both pathways are involved in regulation of the survival of mammalian cumulus/granulosa cells (Kim et al., 1999; Cecconi et al., 2012). In addition, PRL and GH have been shown to inhibit the apoptotic degeneration of bovine cumulus cells surrounding oocytes maturing *in vitro*, with protein kinase C mediating this effect in the case of PRL (Kölle et al., 2003; Lebedeva et al., 2011). Therefore, it seems probable that PRL and GH may eliminate the accelerating action of cumulus cells on abnormal chromosomal modifications in aging bovine oocytes by decelerating cumulus apoptosis and thereby reducing the production of the putative APF (Figure 11). However, other pathways, namely an apoptosis-independent suppression of the APF production or a direct hormonal inhibition of the negative action of APF on bovine oocytes, which can express PRL and GH receptors (Izadyar et al., 2000; Lebedeva et al., 2014b), must not be ruled out.

The findings about activation of the Akt-dependent signaling cascade in bovine aging cumulus-oocyte complexes in response to PRL and GH could explain the opposite pattern of effects of low and high hormonal concentrations on chromosome modifications in oocytes. According to the current knowledge, moderate levels of Akt activity inhibit apoptosis in somatic cells. By contrast, hyperactivation of Akt triggers the cell senescence and apoptotic degeneration by increasing reactive oxygen species (ROS) and suppressing antioxidant enzymes (Los et al., 2009). Thus, PRL and GH at high concentrations might raise the activity of Akt kinase up to critical levels leading to apoptosis and an enhanced production of APF in cumulus cells.

Overall, the findings of the present research point to a possible implication of PRL and GH in regulation of postovulatory aging of bovine oocytes. They suggest that both hormones at physiological concentrations are able to maintain the condensed state of metaphase-II chromosomes by activating intracellular survival pathways in cumulus-oocyte complexes and thereby attenuating negative effects of senescent cumulus cells. Further experiments are needed to clarify interrelations between signal cascades activated by PRL and GH, apoptosis in cumulus cells, epigenetic and chromosome modifications in aging oocytes, and the oocyte competence for the embryonic development. Moreover, an identification of the cumulus-derived aging-promoting factor/factors in the bovine is required.

### Conflict of Interest Statement

The authors declare that the research was conducted in the absence of any commercial or financial relationships that could be construed as a potential conflict of interest.
